# Use of hydroxyurea in French-speaking Sub-Saharan Africa

**DOI:** 10.1007/s00277-024-06180-2

**Published:** 2025-02-05

**Authors:** Jean-Benoît Arlet, Françoise Bernaudin, Indou Deme-Ly, Binta Coulibaly, Anne Corbasson, Ibrahima Diagne, Dapa Diallo, Saliou Diop, Charlotte Eposse, Frédéric Galacteros, Olivier Hermine, Eléonore Kafando, Agnès Lainé, Mariane de Montalembert, Constant Vodouhé, Ahoefa Vovor, Brigitte Ranque, Léon Tshilolo

**Affiliations:** 1 Department of Internal Medicine, Georges Pompidou European Hospital, AP-HP, French National Reference Center for Sickle Cell Anemia, Thalassemia, Other Rare Red Blood Cell Disorders and Erythropoiesis, Paris, and Université Paris-Cité, Paris, France; 2 Pediatric Department Sickle Cell Referral Center, Intercommunal Hospital of Créteil, 94000 Creteil, France; 3 Cheikh Anta Diop University, Dakar, Senegal; 4 Albert Royer National Children Hospital, Dakar, Senegal; 5 Francfort-Sur-Le-Main, Germany; 6 Department of Internal Medicine, Georges Pompidou European Hospital, AP-HP, French National Reference Center for Sickle Cell Anemia, Thalassemia, Other Rare Red Blood Cell Disorders and Erythropoiesis, Paris, France; 7 Cooperation and Pedagogic Innovation, Gaston Berger University of Saint-Louis, Saint-Louis, Senegal; 8 Hematology at the Faculty of Medicine and Odontostomatology of the University of Science, Techniques and Technologies (USTTB), , Bamako, Mali; 9 Center for Research and Control of Sickle Cell Disease (CRLD), Bamako, Mali; 10 Department of Hematology, University Cheikh Anta Diop, Dakar, Senegal; 11 Douala Laquintinie Hospital, Faculty of Medecine and Pharmaceutical Sciences, University of Douala, Douala, Cameroon; 12 Unité Des Maladies Génétiques du Globule Rouge (UMGGR), GHU Henri-Mondor, AP-HP Et U-PEC, Médecine Interne, Paris, France; 13 Department of Hematology, Necker-Enfants Malades Hospital, AP-HP, Paris, France; 14 Laboratory of Hematology, University Joseph Ki-Zerbo, Ouagadougou, Burkina Faso; 15 Institut Des Mondes Africains, Aubervilliers, France; 16 Service de Pédiatrie Générale, Hôpital Necker, Assistance Publique -Hôpitaux de Paris, Paris, France; 17 Association DORYS, Strasbourg, France; 18 Faculty of Health Sciences, Lomé, Togo; 19 Department of Pediatrics, Centre Hospitalier Monkole, Kinshasa, Democratic Republic of Congo

**Keywords:** Sickle cell disease, Africa, French-speaking countries, Guidelines, Hydroxyurea

## Abstract

The hydroxyurea is a save, affordable and essential medicine for sickle cell disease (SCD), reducing painful crises and mortality. To foster the prescription of hydroxyurea for patients with SCD in sub-Saharan African countries, the scientific advisory board of Drep.Afrique, a non-governmental organization dedicated to SCD in Africa, has developed a therapeutic guideline well suited to conditions on the ground. These guidelines answer three essential questions: which patients should be prioritized for treatment with hydroxyurea, which dose should be used, and what follow-up should be implemented in those low-resources countries?

The guidelines are given as in a concise document for easy use by practitioners on the ground, available in English and in French.

**Supplementary Information:**

The online version contains supplementary material available at 10.1007/s00277-024-06180-2.

Sickle cell disease (SCD) is the most prevalent monogenic disorder worldwide; it affects millions of people and around 515,000 live births per year in 2021, mostly in Sub-Saharan Africa (SSA) (79% of the birth) [1]. This recessive condition is due to a single point mutation in the beta-globin gene. The resulting sickle haemoglobin (HbS) polymerizes under low-oxygen conditions. SCD is characterized by chronic haemolytic anaemia, the recurrence of painful vaso-occlusive events (VOE), and acute and chronic damage to multiple organs.

The results of many studies plead in favor of the widespread use of hydroxyurea (HU) in Africa for patients with SCD, as this drug has been shown to dramatically decrease the frequency of VOE, the use of red blood cell transfusions and reduce the mortality rate in this patient population [1–4]. The recent World Health Organization (WHO) African guidance documents (19 June 2024) named “WHO sickle package of intervention for SCD management” clearly states in its conclusion that “all patients in SSA should be offered HU from about the first year of life” [5].

In practice, however, limited access to HU (notably because of the cost to families or supply disruptions) makes it difficult to achieve widespread use in 2025 [6]. Moreover, levels of awareness of HU are generally low among providers and patients in sub-Saharan Africa.

To the best of our knowledge, there are no specific/adapted guidelines on the use of HU to treat patients with SCD in French-speaking countries in Africa, even though about half of the African patients with SCD live in these regions. The ministries of health of Kenya in 2020 [7], of Tanzania in 2021 [8], of Uganda in 2023 [10], and of India in 2023 [10] have published guidelines for hydroxyurea use. A pragmatic hydroxyurea dosing algorithm for low-resource countries was also proposed by the American specialists A. Power-Hays and R. Ware, but indications were not discussed [11]. While these efforts are to be commended, these guidelines are not all consistent in terms of indications, dosage and treatment monitoring.

The non-governmental organization Drep.Afrique was created in June 2018 and is focusing on two essential actions in French-speaking African countries: (i) training the greatest possible number of healthcare professionals in the management of SCD [12] and (ii) providing access to essential drugs in general and HU in particular. The Drep.Afrique scientific advisory board brings together acknowledged African and French specialists in SCD (research scientists, physicians, pharmacists, sociologists, biologists, and nurses), with gender and North/South parity.

Here, the Drep.Afrique scientific advisory board has developed a pragmatic consensus well suited to conditions on the ground and that answers three essential questions: which patients should be prioritized for treatment with HU, which dose should be used, and what follow-up should be implemented?

Following three 2-h meetings (on November 21st, 2023, December 14th, 2023, and February 29th, 2024), the board produced, reviewed and validated the consensus presented here. The objective was to answer the above-mentioned questions in a concise, clear document available in English (below) and in French (Supplemental Fig. 1). We hope that the dissemination of a document for application in Sub-Saharan Africa will enable more SCD patients to be treated—especially those most in need.

## Use of hydroxyurea in French-speaking Sub-Saharan Africa

These guidelines concern patients with sickle cell disease (SCD), children and adults, with SS or S/ß0-thalassemia genotypes.

Ideally, the indication for hydroxyurea (HU) should be discussed with a SCD specialist. This advice can be given remotely and should not delay prescription of the drug in the most severe cases.

## Absolute indications for urgent HU initiation (i.e. immediately upon hospital admission or the discovery of abnormalities)

- After the first episode of acute chest syndrome.
- After a second, severe vaso-occlusive crisis (VOC) in 12 months. A severe VOC is defined as one requiring hospital admission or treatment with intravenous analgesics (whether opioid or not).
- Stroke: transfuse immediately and initiate HU without delay. The HU will be maintained over the long term. Continue, with a red blood cell (RBC) transfusion program, if possible and if transfusion safety conditions are met.
- Transcranial Doppler with a velocity > 200 cm/s: initiate HU (to be maintained over the long term) and, if possible, an RBC transfusion program.
- Acute splenic sequestration: emergency transfusion and the initiation of HU.

From what age can HU be initiated? From the age of 9 months.

## Non-emergency but clear indications

After the patient/parents have been given detailed information about HU and have provided their consent

- Recurrent VOCs with impairment of quality of life and/or family repercussions (school, work, frequent consultations, etc.).
- Severe anemia: a steady-state hemoglobin (Hb) level ≤ 7 g/dL.
- Growth disorders after other causes (notably nutritional or endocrine) have been ruled out.
- Transcranial Doppler with a velocity > 170 cm/s.
- A patient who lives far from an SCD management center.
- Persistent proteinuria caused solely by SCD.

## Prescribing instructions (see algorithm in Supplemental Fig. 2)

The starting dose: 15 mg/kg/day (or 10 mg/kg/day if there are financial restrictions).

- Increase the dose (up to a maximum of 35 mg/kg/day) if clinical effectiveness* has not been achieved after 3 to 6 months.

These increases may be stepwise in increments of 2.5–5 mg/kg at least 2 months apart, after checking on patient compliance and hematologic tolerance.

- Every 6 months or so, adjust the dose according to the child's bodyweight.

*Inefficiency is defined as persistence of VOCs affecting quality of life; one acute chest syndrome; severe VOCs (*defined above*); one ischemic stroke; persistence of accelerated velocity (> 170 cm/s) on transcranial Doppler (if available, in children).

How should HU be taken? The capsule should be taken once a day. It can be dissolved in milk/fruit juice/sauce/yogurt if the patient has difficulty swallowing. Any powder spilled outside the food must be removed immediately, and the dishes as well as the hands of any third party must be washed.

## The minimum level of hematological monitoring

A blood cell count (including hemoglobin, neutrophils and platelets):

- at initiation (month 0).
- at 1 month.
- at 4 months, and then every 3 to 6 months.

Blood counts should be more frequent if the HU dose changes or a history of cytopenia.

## Useful points for patient follow-up

- Upon treatment initiation, note the Hb level, the mean corpuscular volume (MCV) and the neutrophil count (as an absolute value, not a percentage) in the patient’s medical records.
- If the MCV does not increase after the initiation of HU, consider increasing the dose and (above all) check whether the patient is compliant.
- If the neutrophil count is around 2000/mm^3^ (2 G/L), do not increase the dose.
- If the neutrophil count is < 1200/mm^3^ or the platelet count is < 80,000/mm^3^: discontinue HU temporarily for 1 to 2 weeks and resume treatment at a lower dose.
- If the Hb level falls, measure the reticulocyte count (as an absolute value). Lower the HU dose if the reticulocyte count is low (< 80,000/mm^3^) and no other cause of anemia (especially an infection or iron deficiency) is plausible. It is noteworthy that the Hb level usually rises after SCD patients start to take HU.

## Additional information on prescribing HU

- During treatment with HU, continue penicillin V (in children) and folic acid treatments, malaria prevention, and vaccinations.
- Non-infected sickle-cell skin ulcers do not constitute a contraindication to the initiation of HU but their progression must be monitored closely.
- In the event of a proven, severe bacterial infection, delay the initiation of HU treatment. However, it is not necessary to stop HU during long-term course of treatment if an infection is suspected, unless the neutrophil count is <1500/mm^3^.
- **Discontinuation of the treatment:**
  - **Temporally** if cytopenia (see above); pregnancy; difficulty for a man to procreate;
  - **Definitively** if recurrent crises persist 6 months after achieving a target neutrophil count of approximatively 2000/mm3, after careful monitoring of compliance with HU, and after ruling out a deep-seated infection. It may be possible to reintroduce the HU at a later stage in life.

## Areas of prescription uncertainty in Sub-Saharan Africa

Many experts recommend broad, prolonged prescription of HU (i.e. for many years) as soon as SCD (genotypes SS or S/ß°-thalassemia) has been diagnosed after the age of 9 months, without waiting for the onset of complications. It is also integrated in the recent guidelines of the “WHO sickle package of interventions for sickle cell disease management” [5].

This approach has several advantages: the child develops better, has fewer VOCs and strokes and is less likely to experience school absenteeism, transfusion exposure risks, and long-term organ damage. In summary, there is no need to wait for complications.

We have not been able to reach a consensus on this issue because long-term adherence to HU treatment may be difficult if the family has not experienced the impact of a VOC. Indeed, long-term HU therapy and the associated monitoring (blood counts) may place a significant financial burden on the family because no countries do not provides full healthcare coverage. Furthermore, the availability of HU is far from optimal. In some African countries, the wider use of HU might create a risk of shortages for patients who need the drug most.

Although HU dose optimization of up to the maximum hematologically tolerated dose (MTD) is proven to be beneficial in children with SCD (target neutrophil count 2000-4000/mm3), it also runs the risk of creating drug supply disruptions and significantly increases the financial burden on families, who have to pay for close monitoring as well as the higher monthly cost of the drug itself. Moreover, this follow-up can only be provided by prescribers who already have extensive experience of prescribing HU and monitoring it.

In 2025, the systematic, early use of HU, a higher starting dose and the MTD strategy are therefore subject to debate and depend on the country, the center, the family's financial situation, and access to follow-up examinations.

These practical guidelines will certainly evolve as HU becomes more widely available in Africa and as prescribers and families gain experience.

## Supplementary Information

Below is the link to the electronic supplementary material.

Supplementary file1 (DOCX 20 KB)

Supplementary file2 (PDF 1586 KB)

## Data Availability

No datasets were generated or analysed during the current study.
