# Supplementary material for: Use of hydroxyurea in French-speaking Sub-Saharan Africa
Source: Ann Hematol. 2025 Feb 5;104(2):937–41. doi: 10.1007/s00277-024-06180-2 (PMC11971222; doi:10.1007/s00277-024-06180-2)
Supplement: Supplementary file 1 — Supplementary file1 (DOCX 20 KB) [file 277_2024_6180_MOESM1_ESM.docx]

**Figure 2 : Guidelines in French**

**Utilisation de l’hydroxyurée en Afrique Sub-Saharienne Francophone**

***Recommandations du conseil scientifique de Drep.Afrique***

**Préambule**

De très nombreuses études scientifiques plaident pour une utilisation large de l’hydroxyurée (HU) en Afrique car elles ont montré une réduction très importante des crises, du recours aux transfusions globulaires et de la mortalité des patients drépanocytaires traités.

Cependant l’accès limité au médicament, notamment du fait du coût pour les familles ou des ruptures d’approvisionnement, rend, en pratique, difficile sa généralisation en 2025.

Il n’existe pas, à notre connaissance, de recommandations sur la prescription de l’HU dans ce contexte socio-économique particulier dans les pays francophones.

Le conseil scientifique de Drep.Afrique, composé à parité de spécialistes Africains et Français de la maladie (<https://www.drep-afrique.org/equipe/>), propose ici un consensus pragmatique et adapté aux conditions du terrain à travers des réponses à trois questions essentielles : quels patients traiter ? (et surtout qui ne pas oublier de traiter ?) ; à quelle dose ? ;avec quel schéma de surveillance ?

Ces recommandations concernent les patients drépanocytaires enfants et adultes (homozygotes **SS ou S/ß^0^-thalassémiques).**

Idéalement, il convient de discuter de l’indication avec un spécialiste de la drépanocytose. Cet avis peut se prendre en distanciel et ne doit pas retarder la prescription du médicament pour les cas les plus graves.

**Indications formelles et urgentes (à débuter dès l’hospitalisation ou à la découverte des anomalies)**

- Dès le premier syndrome thoracique aigu ;
- Dès la deuxième crise vaso-occlusive (CVO) sévère : nécessitant une hospitalisation ou un antalgique injectable (opioïde ou non) sur les 12 derniers mois ;
- Accident vasculaire cérébral : transfuser en urgence et débuter sans délai l’HU. En plus d’un traitement par HU à long terme, mettre en place un programme transfusionnel, chaque fois que cela est possible et que les conditions de sécurité transfusionnelles le permettent ;
- Doppler transcrânien avec vitesse > 200 cm/s : débuter l’HU (à garder au long cours) et un programme transfusionnel chaque fois que cela est possible ;
- Séquestration splénique aiguë : transfuser en urgence et débuter l’HU.

Quand débuter l’HU ?  Possible dès l’âge de 9 mois

**Indications certaines, non urgentes**

*Après information détaillée sur le produit et accord du patient ou des parents*

- Crises répétées avec altération de la qualité de vie et/ou retentissement familial (école, travail, consultations fréquentes…) ;
- Anémie profonde à l’état basal (taux d’hémoglobine (Hb) ≤ 7 g/dL) ;
- Troubles de croissance après avoir éliminé les autres causes (notamment nutritionnelles ou endocriniennes) ;
- Doppler transcrânien avec vitesse >170 cm/s ;
- Patient vivant loin des centres de soin de gestion des crises ;
- Protéinurie significative et persistante sans autre cause que la drépanocytose.

**Modalités de prescription**

**Dose initiale : 15 mg/kg/jour** (possibilité si nécessaire de débuter à 10 mg/kg/j, si ressources financières limitées).

- Augmentation après 3 à 6 mois si efficacité clinique* non atteinte (jusqu’à un maximum 35 mg/kg/jour).

*L’augmentation peut se faire par paliers de 2,5 mg à 5 mg/kg, espacés d’au moins 2 mois, après avoir vérifié l’observance et la tolérance hématologique.*

- Adapter environ tous les 6 mois la dose au poids évolutif des enfants

** L’inefficacité est définie comme la persistance de CVO répétées altérant la qualité de vie ; la survenu d’un syndrome thoracique ; de CVO sévères (comme défini antérieurement) ; d’une accélération des vitesses au Doppler transcrânien ( >170 cm), si l’examen est disponible.*

**Comment le prendre ? En une fois par jour.** En cas de difficulté à l’avaler, la gélule (lorsque c’est cette formulation) peut être ouverte et son contenu dissout dans du lait/du jus de fruit/de la compote/ du yaourt. Toute poudre répandue hors de l’aliment devra être immédiatement éliminée ; la vaisselle et les mains de la tierce personne nettoyées.

**Suivi hématologique minimal**

Un hémogramme avec numération des polynucléaires neutrophiles (PNN) et des plaquettes :

- A l’initiation (M0)*;*
- Puis à 1 mois ;
- Puis à 4 mois, puis tous les 3 à 6 mois.

Faire des hémogrammes plus rapprochés en cas de changement de dose ou si antécédent de cytopénie.

*Paramètres utiles pour la surveillance :*

- *à l’initiation de l’HU, bien noter dans le dossier du patient le taux d’Hb, le volume globulaire moyen (VGM) et la concentration des PNN (en valeur absolue et non en pourcentage) ;*
- *si le VGM n’augmente pas sous HU, évoquer une dose insuffisante ou, surtout, un défaut d’observance ;*
- *si la concentration en PNN est autour de 2000/mm*^3^ *(2 G/L): ne pas augmenter la dose.*
- *si la concentration en PNN <1200/mm*^3^ *(ou 1,2 G/L) ou en plaquettes < 80 000/mm*^3^*(ou 80 G/L), suspendre pour 1 à 2 semaines et reprendre à plus faible dose ;*
- *si aggravation de l’anémie, doser les réticulocytes (en valeur absolue). Diminuer la dose d’HU si les réticulocytes sont bas (< 80 000/mm*^3^*) et qu’il n’y a pas d’autre cause d’anémie (notamment infection et carence en fer). Il est à noter que l’Hb augmente généralement chez les patients drépanocytaires traités par HU.*

**INFORMATIONS COMPLEMENTAIRES**

- Poursuivre la pénicilline V (chez l’enfant), l’acide folique, la prévention de la malaria et les vaccinations en plus de l’HU.
- Les ulcères cutanés drépanocytaires non infectés ne sont pas une contre-indication à l’initiation de l’HU mais nécessitent une surveillance rapprochée de leur évolution.
- En cas d’infection bactérienne grave avérée, retarder le début du traitement. Lors d’une prise au long cours, la suspicion d’une infection ne nécessite pas l’arrêt de l’HU, sauf si les PNN sont <1500/mm^3^.
- **Arrêt du traitement :**
  - **Transitoire** en cas de cytopénies (voir plus haut); de grossesse; de difficulté de procréer pour un homme;
  - **Définitif** si des CVO persistent après 6 mois de traitement bien pris et à dose optimisée pour atteindre une concentration PNN autour de 2000/mm^3^, après avoir vérifié la compliance et éliminé une infection profonde. L’HU peut être réessayer plus tard dans sa vie.

**Zones d’incertitude de la prescription en Afrique Sub-saharienne**

De nombreux auteurs recommandent la prescription large et prolongée pendant des années dès que le diagnostic de drépanocytose SS ou S/ß°-thalassémie est établi (après 9 mois de vie), sans attendre l’arrivée des complications. C’est aussi les recommandations de l’OMS pour l’Afrique dans un récent rapport de juin 2024.

Les avantages de l’approche sont : permettre un meilleur développement de l’enfant, réduire les crises, les AVC, l’absentéisme scolaire, les risques d’exposition transfusionnelle, et les lésions chroniques d’organes… en résumé ne pas attendre la survenue de complications.

Nous n’avons pas pu dégager un consensus à ce sujet car l’adhérence au traitement sur le long terme pourrait être difficile si les familles n’ont jamais expérimenté l’impact des crises. De plus, il existe un fardeau important sur la famille lié au coût du traitement de l’HU sur le long terme et de sa surveillance (hémogramme), tant qu’il n’y a pas dans les pays un système de prise en charge à 100%. La disponibilité est aussi loin d’être optimale. L’utilisation très large pourrait créer un risque de pénurie pour des patients jugés prioritaires.

Enfin, l'utilisation, chez tous les patients, d'une dose maximale tolérée (DMT) sur le plan hématologique (objectif des PNN entre 2000 et 4000/mm^3^), même si l’efficacité de cette stratégie a bien été démontrée chez les enfants drépanocytaires, est aussi à risque de créer une pénurie et augmente nettement le coût pour les familles (non seulement du traitement, mais aussi de sa surveillance). Par ailleurs, cela ne peut être envisagé que par des prescripteurs ayant déjà une large expérience de la prescription de l'HU et de sa surveillance.

Ainsi, l’utilisation systématique et précoce de l’HU, une dose d’attaque plus élevée, et la stratégie de la DMT doit, en 2025, se discuter selon les pays, les centres, les possibilités financières des familles et de l’accès aux examens de suivi.

Ces recommandations seront certainement évolutives en fonction de la diffusion de l’HU en Afrique et de l’expérience croissante des prescripteurs et des familles.
