# Supplementary material for: Use of hydroxyurea in French-speaking Sub-Saharan Africa
Source: Ann Hematol. 2025 Feb 5;104(2):937–41. doi: 10.1007/s00277-024-06180-2 (PMC11971222; doi:10.1007/s00277-024-06180-2)
Supplement: Supplementary file 2 — Supplementary file2 (PDF 1586 KB) [file 277_2024_6180_MOESM2_ESM.pdf]

A. Minimum baseline laboratory testing: hemogram with neutrophils count

DREP AFRIQUE

2025 Guidelines

Starting dose of hydroxyurea (HU): 15 mg/kg/day
Once a day

Blood counts (Hb, MCV, neutrophils count) at 1 months (m), then every 3-6 m

Clinical effectiveness every 3-6 month

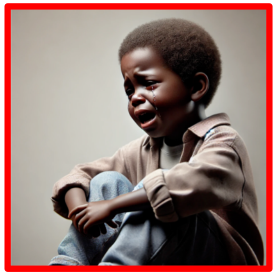

- Persistent VOCs
- Acute chest syndrome
- Severe VOCs or Stroke
- High velocity on Doppler

Increase dose
2.5-5 mg/kg every 2 months
if neutrophils >2000/mm^3
(maximal dose 35 mg/kg/day)

Good clinical response

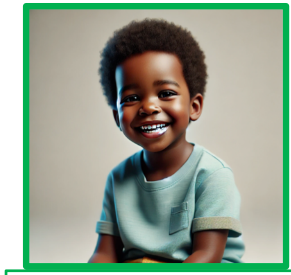

No change
Blood count/3-6 m

Adjust the dose
to the bodyweight

Toxicity thresholds

Neutrophils <1200/mm^3
Platelet count <80000/mm^3
Hb <5 g/dL and retic. <80000/mm^3

Stop HU for 1 to 2 weeks
Resume HU at a lower dose

Discontinuation criteria:

Cytopenia, pregnancy,
Non-compliance

Persistent VOCs with neutrophils ≈ 2000/mm^3

B. Bilan biologique minimum: hémogramme avec numération des neutrophiles

DREPAFRIQUE
2025 Guidelines

Dose initiale de l'hydroxyurée: 15 mg/kg/day une fois par jour

Hémogramme (Hb, VGM, neutrophiles) à 1 mois (m), puis tous les 3-6 m

Seuils de toxicité
Neutrophiles <1200/mm^3
Plaquettes <80000/mm^3
Hb <5 g/dL avec r"tic. <80000/mm^3

Stop HU pendant 1 à 2 semaines
Reprende l'HU à dose plus faible

Efficacité clinique tous les 3-6 mois

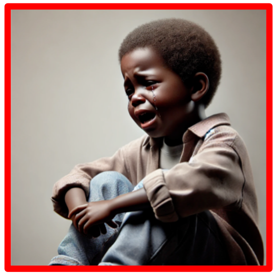

- CVOs persistantes
- Syndrome thoracique aigu
- CVOs sévère ou AVC
- Vitesses accélérées au DTC

Augmenter la dose
2,5-5 mg/kg tous les 2 mois
si neutrophiles >2000/mm^3
(dose maximale 35 mg/kg/day)

Bonne réponse clinique

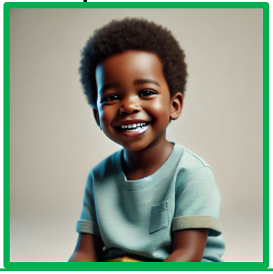

Pas de changement
Hémogramme/3-6 m

Adapter la dose au poids

Critères d'arrêt:
Cytopénie, grossesse,
Non-compliance
CVOs récurrentes avec neutrophiles ≈ 2000/mm^3
